# Association Between Electronic Health Record Health Maintenance Topic and Lung Cancer Screening Completion in a Resident-Run Clinic

**DOI:** 10.7759/cureus.116578

**Published:** 2026-09-20

**Authors:** Lawrence J Stawkowski, Caty Davenport, Kathryn Zaffiri, Amy Slenker

**Affiliations:** 1 Internal Medicine, Lehigh Valley Health Network, Allentown, USA

**Keywords:** clinical decision support, electronic health record, low-dose computed tomography, lung cancer screening, primary care, quality improvement, resident education

## Abstract

Introduction: Lung cancer screening (LCS) with low-dose computed tomography (LDCT) reduces lung cancer-specific mortality in high-risk populations, yet screening rates remain low nationwide. Electronic health record (EHR) health maintenance tracking may support guideline-concordant screening, but implementation varies across outpatient settings.

Objective: To evaluate the association between manually adding the LCS health maintenance topic in the EHR and completion of LDCT screening in a resident-run outpatient clinic.

Methods: This retrospective cross-sectional study evaluated adult patients at a resident-run Federally Qualified Health Center Look-Alike clinic who met age and smoking history criteria for LCS in October 2024. Patients were categorized based on whether the LCS health maintenance topic was manually added within the Epic EHR and whether LDCT screening had been completed. Screening completion rates were compared using a chi-square test.

Results: Among 305 eligible patients, 130 (43%) completed LDCT screening. Of screened patients, 96 (74%) had the LCS health maintenance topic added, compared with 86 (49%) of 175 (57%) unscreened patients. Adding the LCS health maintenance topic was significantly associated with LDCT screening completion (p<0.001).

Conclusion: Having the LCS health maintenance topic activated in the EHR was associated with higher rates of LDCT screening completion in a resident-run clinic compared with not having the LCS health maintenance topic activated. These findings support the role of having the LCS health maintenance topic added in the EHR as a practical strategy to reinforce preventive care delivery in outpatient practice.

## Introduction

Lung cancer remains the leading cause of cancer-related mortality in the United States [1]. Screening with low-dose computed tomography (LDCT) has been shown to reduce lung cancer-specific mortality by 16%-20% in high-risk populations, leading to national guideline recommendations for screening [1]. The 2021 US Preventive Services Task Force (USPSTF) guidelines expanded screening eligibility to individuals aged 50-80 years with a ≥20 pack-year smoking history. This expanded eligibility to include younger smokers with lower pack-year histories to address disparities in screening access [1,2]. Despite these evidence-based recommendations, national lung cancer screening (LCS) rates remain poorly optimized. Complex implementation barriers persist, including clinician time constraints during brief encounters, complex documentation requirements, low patient awareness, and systemic challenges unique to underserved populations, such as lack of transportation, cost, and limited access to thoracic imaging centers [3]. Currently, fewer than one in five eligible individuals nationwide remain up to date with screening [3,4]. Primary care providers in resident-run clinics face considerable time constraints, with estimates suggesting that over seven hours per day would be required to fulfill all recommended preventive services alone [4,5].

Electronic health record (EHR) systems incorporate preventive care reminders and Health Maintenance topics to support guideline-concordant care [2]. However, inconsistent implementation of these tools may contribute to missed screening opportunities, particularly in safety-net and resident-run clinics.

At our internal medicine resident-run outpatient clinic, LCS health maintenance topics were not automatically added for eligible patients, requiring providers to manually add the LCS health maintenance topic to the patient’s chart. We evaluated whether the presence of an LCS health maintenance topic in the EHR was associated with increased completion of LDCT screening.

This work was previously presented as an abstract at the American College of Chest Physicians (CHEST) Annual Meeting, Chicago, IL, in October 2025.

## Materials and methods

This retrospective cross-sectional study was conducted using the Epic EHR system (Epic Systems Corporation, Verona, WI, USA) at an outpatient internal medicine resident-run Federally Qualified Health Center Look-Alike clinic in Allentown, PA, USA. Patient electronic records were cross-sectionally evaluated during a defined one-month data collection period in October 2024.

Study design and framework

This study was reported in accordance with the Strengthening the Reporting of Observational Studies in Epidemiology (STROBE) guidelines for cross-sectional studies. The study was determined to be exempt from formal Institutional Review Board (IRB) approval as it utilized de-identified retrospective data for clinical workflow evaluation. Screening eligibility was based on the hospital network policy and defined as patients aged 50-77 years with at least a 20 pack-year smoking history who were current smokers or former smokers who had quit within the previous 15 years. This was in comparison with the CHEST and USPSTF guidelines, which include patients 50-80 years old [1,6,7]. Patients were categorized based on the following criteria: whether the LCS health maintenance topic had been added to the EHR and whether LDCT or chest CT had been completed in the previous year.

Screening completion was defined as any patient meeting the inclusion criteria who had documented completion of an LDCT or chest CT scan as of October 2024. The LCS health maintenance topic is defined as a type of automatic screening tracker that can be added to a patient’s EHR in Epic for specific recommended preventive and screening tests. The tool acts as a point-of-care clinical decision support (CDS) tool present in the sidebar of the Epic EHR when reviewing the patient’s chart.

Setting and population

Patients from the Allentown, PA, outpatient clinic were identified through EHR-based reports using the Epic SlicerDicer tool. The Epic SlicerDicer tool is used to analyze and aggregate data through an easy-to-use interface. A total of 7,115 patients were initially screened for eligibility. Data were collected in October 2024.

Inclusion criteria

Patients were included if they met the following criteria: age 50-77 years, ≥ 20 pack-year smoking history, and current smokers or former smokers who quit within the past 15 years.

Exclusion criteria

Any patient who did not meet the inclusion criteria.

Data collection

Patients were identified using the Epic SlicerDicer tool to ensure a standardized and reproducible cohort identification process. Within the "Patients" data model, the cohort was filtered by the specific internal medicine resident clinical department. The "Lung Cancer Screening" Health Care Topic slice was utilized to separate patients meeting the study's inclusion criteria: ages 50-77 years, a ≥20 pack-year smoking history, and currently smoking or having quit within the past 15 years. Data were collected in October 2024.

Statistical analysis

Data analysis was performed using Microsoft Excel (Microsoft Corporation, Redmond, WA, USA). Screening completion rates were compared between patients with and without the added LCS health maintenance topic using a chi-square test. A p-value of < 0.05 was considered statistically significant. The following formula was used: \[ \chi^2 = \sum \frac{(O_i-E_i)^2}{E_i} \] where (Oi) represents the observed frequency and (Ei) represents the expected frequency.

## Results

A total of 305 patients met the inclusion criteria. Of these, 148 (48.5%) were female, and 157 (51.5%) were male. Of the 148 female patients, 66 (44.6%) completed screening, compared to 64 (40.8%) of 157 male patients. Data on individual clinician documentation of patients explicitly declining screening or the precise incidence of positive lung nodules on completed thoracic imaging were not structurally extracted in this report.

Overall, 130 patients (42.6%) completed LDCT screening. Among screened patients, 96 (73.8%) had an LCS health maintenance topic added in the EHR. In contrast, among the 175 (57.4%) patients who had not completed screening, 86 (49.1%) had the LCS health maintenance topic added, as shown in Table 1.

**Table 1:** Baseline characteristics of eligible patients (LDCT) LDCT, low-dose computed tomography

Characteristic	Value
Total cohort	305 (100%)
LDCT screen	
Screened	130 (42.6%)
Unscreened	175 (57.4%)
Sex	
Female	148 (48.5%)
Male	157 (51.5%)

Screening completion differed significantly by having the LCS health maintenance topic added, with higher completion rates among patients with the LCS health maintenance topic added compared with those without it (Table 2). This association is visually demonstrated in Figure 1.

**Table 2:** LDCT completion by EHR health maintenance topic added versus not added OR=2.92 (95% CI: 1.77-4.82); x^2^=18.06, P<0.001; Phi (Φ)=0.24. LCS, lung cancer screening; LDCT, low-dose computed tomography; EHR, electronic health record; OR, odds ratio

EHR LCS health maintenance topic added versus not			
	LDCT completed	LDCT not completed	Total
LCS health maintenance topic added	96	86	182
LCS health maintenance topic not added	34	89	123
Total	130	175	305

**Figure 1:**
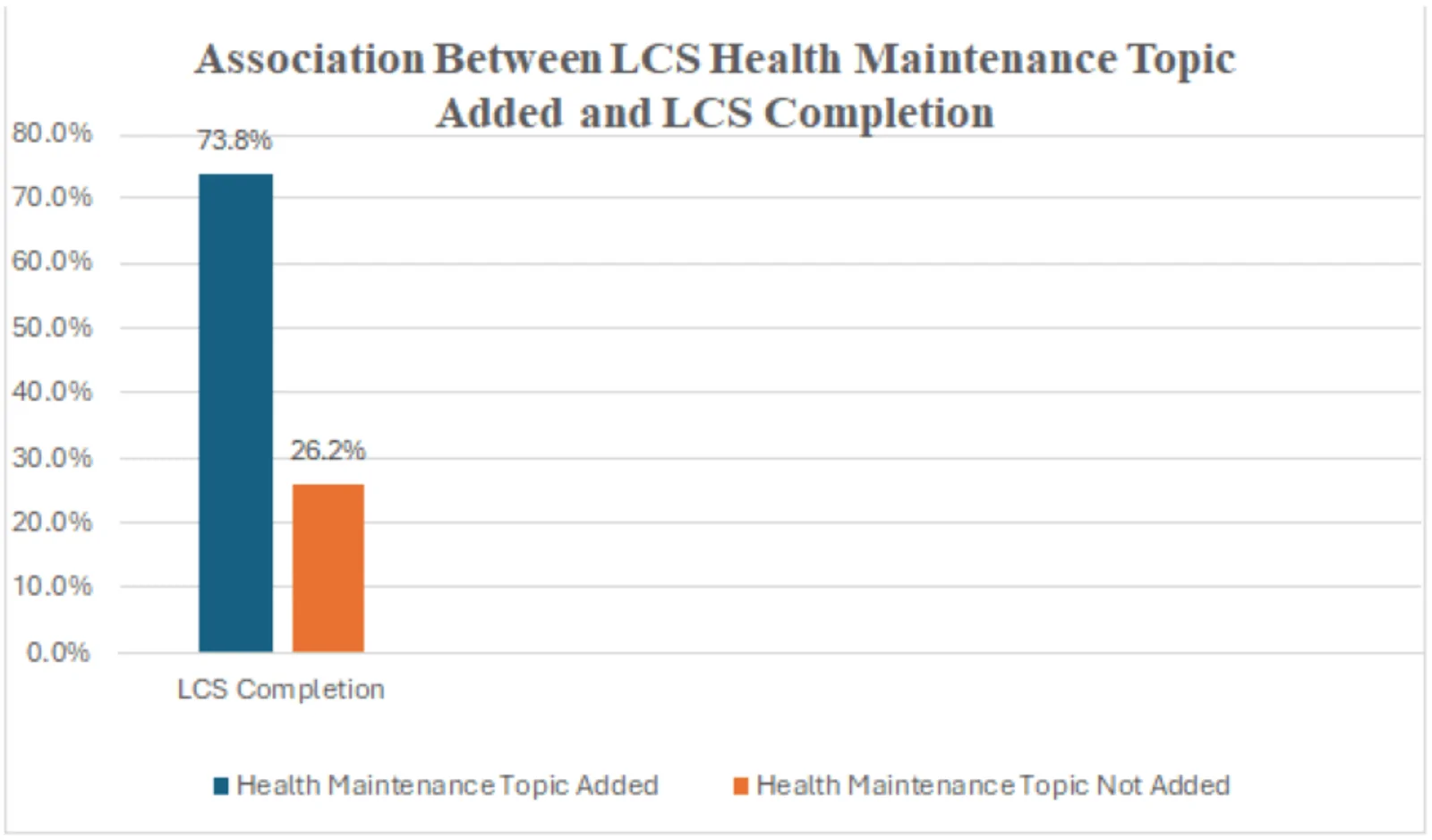
Association between LCS health maintenance topic added and LCS completion LCS, lung cancer screening

Having the LCS health maintenance topic added was significantly associated with higher rates of LDCT screening completion (χ²=18.06, p<0.001). Patients with the LCS health maintenance topic added had nearly three times higher odds of completing LDCT screening compared with those without the topic added (odds ratio (OR)=2.92, 95% CI: 1.77-4.82; Phi [Φ]=0.24, indicating a moderate effect size).

## Discussion

The addition of the LCS health maintenance topic functions as a "digital nudge" within the choice architecture of the EHR, making the screening requirement salient to the clinician during high-acuity encounters [8,9]. By automating the identification of eligible patients at the point of care, these tools reduce the cognitive load on resident physicians, mitigating clinical inertia [10]. The fact that nearly half of unscreened patients had the LCS health maintenance topic underscores that digital tools are an essential trigger but cannot fully overcome patient-level barriers such as transportation, cost, and the complexities of shared decision-making [11-13].

Adding the LCS health maintenance topic represents a low-cost and scalable strategy that may help address provider- and system-level barriers to LCS. Given persistently low national screening uptake, such tools may be particularly useful in outpatient practices with competing clinical demands and support the use of EHR LCS health maintenance topics as a system-level tool to reinforce guideline-concordant preventive care [2-4].

Furthermore, this study demonstrates an association rather than causation. The presence of the LCS health maintenance topic may reflect increased provider awareness, patient engagement, or visit frequency rather than a direct effect of the added LCS health maintenance topic itself.

Limitations

This study has several limitations inherent to its design and setting. First, the cross-sectional nature of the data precludes the assessment of temporal relationships or direct causality. Crucially, because patient records were evaluated cross-sectionally, we cannot establish whether the Health Maintenance topic was manually added prior to or after LDCT completion. While a significant association was found, we cannot definitively conclude that activated LCS health maintenance topics caused screening completion, as the presence of the active topic may reflect general clinician engagement, concurrent documentation, or unmeasured factors-such as resident enthusiasm, patient health literacy, or individual clinician “buy-in.”

Second, there are potential measurement and documentation biases. Because this was a retrospective review of a single EHR system, screenings completed at outside facilities may not have been captured, potentially underestimating the true completion rate. Furthermore, the inclusion criteria counted both dedicated LDCT and standard chest CT scans as successful screening. For patients with frequent hospitalizations requiring diagnostic thoracic imaging, this may overrepresent “guideline-concordant” screening.

Additionally, this study is limited by the parameters extracted during the retrospective chart review. We did not structurally aggregate data regarding the incidence of positive lung nodules or suspicious CT findings within the screened cohort, nor did we track instances where eligible patients explicitly declined screening after a discussion with their provider. Consequently, we cannot differentiate between failure of provider tracking and proactive patient refusal, which remains a critical target for future qualitative quality improvement initiatives.

Finally, the study did not account for provider-level variables, such as visit frequency or individual variation in how often residents accessed the “Health Maintenance” tab. As a single-center study conducted within a resident-run safety-net clinic, these findings reflect the unique workflow and infrastructure of a training environment and may have limited generalizability to private practice or non-academic settings.

## Conclusions

Adding the LCS health maintenance topic within the EHR was significantly associated with higher rates of LDCT completion in a resident-led outpatient setting. These findings suggest that EHR-integrated Health Maintenance tools may function as a passive nudge for clinicians, potentially lowering the cognitive load required to identify high-risk patients during busy clinical encounters. Unlike traditional outreach campaigns that demand consistent staff effort, maintaining an active Health Maintenance topic provides a continuous, low-resource safety net for preventive oncology.

Identifying this association provides a helpful evidentiary foundation for healthcare systems considering multi-level strategies, such as automated patient messaging or nurse-led screening coordination. Because standard EHR platforms are utilized across most graduate medical education training sites, optimizing these existing digital tools represents a potentially low-cost strategy for healthcare systems seeking to reinforce screening delivery in underserved populations.
